# Thioredoxin and Glutaredoxin Systems Antioxidants Special Issue

**DOI:** 10.3390/antiox8030068

**Published:** 2019-03-18

**Authors:** Jean-Pierre Jacquot, Mirko Zaffagnini

**Affiliations:** 1 Université de Lorraine, Inra, IAM, F-54000 Nancy, France; 2 Laboratory of Molecular Plant Physiology, Department of Pharmacy and Biotechnology, University of Bologna, via Irnerio 42, 40126 Bologna, Italy; mirko.zaffagnini3@unibo.it

The special issue on Thioredoxin and Glutaredoxin systems (http://www.mdpi.com/journal/antioxidants/special_issues/Thioredoxin_and_Glutaredoxin_Systems) was initiated in response to solicitations from Antioxidants after discussing with colleagues at two successive redox meetings sponsored by European Molecular Biology Organization (EMBO) and held in July 2017 in Moscow/St. Petersburg (http://redox.vub.ac.be/events/embo-redox-biology-conference.html) and in September of the same year in San Feliu de Guixols (Spain) (http://meetings.embo.org/event/17-thiol-ox). We could then submit the idea to long time collaborators and redox friends but also to other colleagues with whom we had the chance to get in touch with at these meetings. In general, although Antioxidants is a rather recent creation and its credentials were at the time not so well known, the idea of participating in a special issue was very well received and many of the contacted colleagues have answered positively. Of course, as our background is in plant sciences, this special issue mostly contains papers dealing with oxygenic phototrophs but other experimental model organisms are also addressed (bacteria, mammals, zebrafish, etc.). Overall the special issue contains 16 papers, 12 of those reporting experimental research data, and 4 others being more review-like although some of them also contain original bioinformatics data. 

The two volume editors (J.P.J. and M.Z.) wish to testify that the reviewing process has been done in a very professional way by Antioxidants. We have been phased out of the few papers that presented a conflict of interest, but asked to give a final approval for those. All papers were reviewed by at least two international experts, very often three and more rarely four. Occasionally the participants were asked to cross review papers of the special issue but on average this happened quite rarely. This very thorough evaluation system has helped improve the quality of several of the papers by pointing out some weaknesses that were fixed in a second or third round of evaluation.

Thus overall, we are very pleased with the outcome of this editorial effort and we wish here to give a brief summary of its content and most exciting features. 

The first article is a review by Sophie Vriz and colleagues [1]. It deals with Reactive Oxygen Species (ROS) signaling, the very dynamic variation of those species and the morphogenetic, embryogenesis, regeneration, and stem cell differentiation consequences of these molecules. Of course the interaction with reducing molecules as those of the thioredoxin (TRX) and glutaredoxin (GRX) systems can modulate the oxidant signaling. As Vriz and colleagues have extensive experience with zebrafish this experimental model is especially well treated (most other papers deal essentially with bacteria and plants). The two next papers by the laboratories of Pascal Rey and Bertrand Friguet describe properties of methionine sulfoxide reductases (MSRs, enzymes that are able to reduce methionine sulfoxide back to methionine. In the context of increased generation of oxidant molecules, damages can occur on macromolecules including lipids and proteins and thus the function of MSRs is very important in the cell. More precisely Rey and Tarrago [2] describe the relative properties of MSRA and MSRB in plants and the interplay of MSRs with Ca^2+^- and phosphorylation-dependent cascades, thus transmitting ROS-related information in transduction pathways. Lourenço dos Santos, Petropoulos and Friguet [3] discuss the properties of MSRs essentially in bacteria but also detail the generation of cysteine sulfenate (-SOH) leading to the buildup of disulfide bonds that can be reduced back to dithiols via the TRX and GRX systems. 

The next paper by Dreyer and Dietz [4] discusses the regulatory network of the cell leading to cold stress adaptation and provides short-term transcriptome and metabolome analyses that help understand the physiological responses of plants to cold adaptation. Undeniably, in agronomy the resistance of plants to cold is essential for maintaining a high yield required for animal and human nutrition. 

The next paper in the series is a contribution by Monica Balsera and colleagues [5]. Buey, Schmitz, Buchanan and Balsera report the structure of an nicotinamide adenine dinucleotide phosphate reduced (NADPH) TRX reductase (NTR) from an archaeon producing methane, *Methanosarcina mazei*. Interestingly, the protein crystallizes in the apo form lacking flavin adenine dinucleotide (FAD). The apo NTR displays the same dimeric head to tail organization than previously characterized NTRs despite lacking the flavin coenzyme. They discuss the significance of this weaker FAD binding compared to NTR from other organisms including bacteria, animals and plants. 

The next six papers discuss structural and physiological properties of TRXs and GRXs in plants and bacteria. The first of these by the Frendo group [6] discusses the interactions between *Medicago truncatula*–*Sinorhizobium meliloti* which are extremely important in nitrogen fixation for leguminous plants. The symbiotic interaction leads to the formation of a new organ, the root nodule, where a coordinated differentiation of plant cells and bacteria occurs. The crucial role of glutathione in redox balance and sulfur metabolism is presented. They also highlight the specific role of some TRX and GRX systems in bacterial differentiation. Transcriptomics data concerning gene encoding components and targets of TRX and GRX systems in connection with the developmental step of the nodule are considered in this contribution. The paper by Mariam Sahrawy and colleagues [7] follows an interesting approach that has not been used thus far: determining by proteomics the relative abundance of a large panel of proteins in plants lacking either TRX *f* or TRX *m*. Their results revealed a quantitative alteration of 86 proteins and demonstrate that the lack of both the *f*- and *m*-type TRXs have diverse effects on the proteome. Most of the differentially expressed proteins fell into the categories of metabolic processes, the Calvin–Benson cycle, photosynthesis, response to stress, hormone signaling, and protein turnover. Photosynthesis, the Calvin–Benson cycle and carbon metabolism are the most affected processes. Notably, a significant set of proteins related to the answer to stress situations and hormone signaling were affected. Overall, this suggests that the TRX systems may regulate transcription and translation in plants. 

The paper by Stéphane Lemaire et al. [8] reports the crystal structure of TRX *f*2 from *Chlamydomonas reinhardtii*. The systematic comparison of its atomic features to other *f*-type TRXs reveals a specific conserved electropositive crown around the active site, complementary to the electronegative surface of their targets. They postulate that this surface provides specificity to the various type of TRX. The following article of the Javier Florencio group [9] provides information about TRX C, an atypical TRX present exclusively in cyanobacteria. TRX C has a modified active site (WCGLC) instead of the canonical (WCGPC) present in most TRXs and is not active in the classical TRX in vitro tests. Nevertheless, the *ΔtrxC* mutant, although growing at similar rates to WT in all conditions tested, showed an increased carotenoid content especially under low carbon conditions. Their data suggest that TRX C might have a role in regulating photosynthetic adaptation to low carbon and/or high light conditions. Marchand et al. [10] provide a refined 3D structure of *C. reinhardtii* TRX *h*1 in the reduced and oxidized form as well as the one of cysteine mutants. This paper also features data concerning the p*K*_a_ values of both catalytic cysteines by means of iodoacetamide-based mass spectrometry analysis. The next contribution by the Nicolas Rouhier group [11] describes physico-chemical and catalytic properties of the poorly characterized mitochondrial TRX *o*. Very interestingly, they show for the first time that this isoform can in vitro bind iron sulfur centers (ISCs) of the [4Fe-4S] type likely ligated by the classical catalytic cysteines present in the conserved WCGPC signature. This situation is somewhat similar to those of various GRXs which also bind ISCs in a homodimer although the nature of the center is different. Remarkably, their results suggest that a novel regulation mechanism may prevail for mitochondrial *o*-type TRXs, possibly existing as a redox-inactive Fe–S cluster-bound form that could be rapidly converted in a redox-active form upon cluster degradation under specific physiological conditions. NFU proteins could be target of the catalytically active form. It remains to be seen whether the ISC-replete form could be involved in ISC transfer/assembly as suggested for GRXs in many species and sub-compartments. 

In the next four papers, we now turn our attention to target enzymes of the TRX systems in plants. Yoshida and Hisabori [12] investigate the rate limiting step of enzyme light activation in chloroplasts. They found that the catalytic subunit of ferredoxin:TRX reductase (FTR) and *f*-type TRX are rapidly reduced after the drive of reducing power transfer, irrespective of the presence or absence of their downstream target proteins. By contrast, three target proteins, fructose 1,6-bisphosphatase (FBPase), sedoheptulose 1,7-bisphosphatase (SBPase), and RuBisCO activase (RCA) showed different reduction patterns; in particular, SBPase was reduced at a low rate. The in vivo study using *Arabidopsis* plants showed that the TRX family is commonly and rapidly reduced upon high light irradiation, whereas FBPase, SBPase, and RCA are differentially and slowly reduced. Among the GRX targets is cytosolic isocitrate dehydrogenase (cICDH), the activity of which is controlled by S-nitrosylation upon nitrosoglutathione (GSNO) treatment as shown by Reichheld and colleagues [13]. In particular, they have shown that GRXs are able to rescue the GSNO-dependent inhibition of cICDH activity, suggesting that they can act as a denitrosylation system in vitro. They observe that the GRX system, contrary to the TRX system, is able to remove S-nitrosothiol adducts from cICDH and they have specified on which specific cysteine this is occurring. The report by Vanacker et al. (Emmanuelle Issakidis lead author) investigates the redox regulation of monodehydroascorbate reductase (MDHAR) [14]. They found that the activity of leaf extracted or the recombinant plastidial *Arabidopsis thaliana* MDHAR isoform 6 was specifically and strongly increased by reduced TRX *y*, and not by other plastidial TRXs. In addition, TRX *y* mutant plants showed reduced stress tolerance in comparison with wild-type (WT) plants that correlated with an increase in their global protein oxidation levels. The last of the papers dealing with redox regulated enzymes provides results concerning chlorophyll biosynthesis. It is well known that chlorophyll synthesis requires light and one key regulatory enzyme is aminolevulinate dehydratase (ALAD). Berhanrd Grimm and colleagues [15] show that this enzyme interacts with TRX *f*, TRX *m* and NTRC in chloroplasts. The reduced and oxidized forms of ALAD differed in their catalytic activity and they conclude that (i) deficiency of the reducing power mainly affected the in planta stability of ALAD; and (ii) the reduced form of ALAD displayed increased enzymatic activity. 

The last paper of this special issue is by Nicolas Navrot et al. [16]. This contribution of the Svensson lab has investigated the biotechnological potential of the NTR/TRX system (NTS), and especially of NTR, as a useful tool in baking for weakening strong doughs, or in flat product baking. They have shown that the barley NTS is capable of remodeling the gluten network and weakening bread dough.

In conclusion, we believe that this special issue had indeed provided new information about a number of proteins involved in the redox regulatory networks, either oxidants, reductants, TRXs, or targets. Of course, we would have liked to cover additional topics, in particular the field of peroxiredoxins and glutathione peroxidases was not addressed at all. Likewise, two recent papers reported on the interesting diversity of protein disulfide isomerases in plants [17] and this will certainly require a more thorough investigation in the future. Many other examples could be provided for sure, so there are many opportunities for producing future special issues as highlighted by the recent paper uncovering the importance of Cys-based redox mechanisms in plant development, growth and adaptation to stress [18].
